# Improving therapeutic potential of GDNF family ligands

**DOI:** 10.1007/s00441-020-03256-z

**Published:** 2020-07-28

**Authors:** Pia Runeberg-Roos, Richard D Penn

**Affiliations:** 1 Institute of Biotechnology, University of Helsinki, PB 56 (Viikinkaari 5D), FIN-00014 Helsinki, Finland; 2 Present Address: Nanoform Finland, Viikinkaari 4, FI-00790 Helsinki, Finland; 3 Rush Medical School, Chicago, IL USA; 4 University of Illinois at Chicago, Chicago, IL USA

**Keywords:** GDNF family ligands, Parkinson’s disease, Cell biology, NRTN

## Abstract

The last decade has been a frustrating time for investigators who had envisioned major advances in the treatment of Parkinson’s disease using neurotrophic factors. The first trials of glial cell line–derived neurotrophic factor for treating Parkinson’s disease were very promising. Later blinded control trials were disappointing, not reaching the predetermined outcomes for improvement in motor function. Consideration of the problems in the studies as well as the biology of the neurotrophins used can potentially lead to more effective therapies. Parkinson’s disease presents a multitude of opportunities for the cell biologist wanting to understand its pathology and to find possible new avenues for treatment.

## Introduction: Parkinson’s disease and how cell biology can help

Parkinson’s disease presents a multitude of opportunities for the cell biologist wanting to understand its pathology and to find possible new avenues for treatment. As pointed out in a recent review, Parkinson’s disease is a neurological disorder with evolving layers of complexity (see Kalia and Lang 2015 for an excellent clinical summary). The most easily recognized aspects of the disease are the motor signs of slow movement, muscle rigidity, tremor, and postural instability. These are associated with loss of dopaminergic neurons in the substantia nigra and the accumulation of Lewy bodies composed of synuclein in nerve cells. Nonmotor symptoms such as constipation and depression predate clinical motor signs and then, late in the disease, cognitive disabilities increase. These changes are due to non-dopaminergic cell damage. Dopamine replacement works dramatically for the initial motor deficiencies and a strong positive response helps to make the diagnosis of the disease. Unfortunately, as the disease progresses, this treatment becomes less effective and produces major side effects. Deep brain stimulation can significantly improve motor symptoms and reduce some of the side effects of oral drug treatment, but it does not change the course of the disease.

The complexity of the signs and symptoms of Parkinson’s disease is mirrored by the complexity of the many environmental and genetic factors that have been shown to be associated with its development. The growing list of environmental factors that increase risk, such as pesticide exposure, head injury, and rural living, is matched by the enlarging number of genetic risk factors that have been discovered. A few identified genes put patients at high risk for developing the disease and have been tied to its physiology. However, in the vast majority of sporadic cases, 90% plus, genetic causes have not been found or have a very small association (Kalia and Lang 2015).

An example of the importance of novel cell biology approaches for finding new treatments is the recent work on induced pluripotent stem cells (iPSCs) that can be directed into dopaminergic cell types. Using such cells taken from Parkinson patients with the sporadic form, no definitive genetic abnormalities were demonstrated. However, these negative findings are not true for the 10% of sporadic cases of young-onset Parkinson’s disease, ages 21 to 50 (Laperle et al. 2020). Dopaminergic iPSCs generated from these younger patients show increases in synuclein and phosphorylated protein kinase C alpha and reduced levels of liposomal membrane proteins such as LAMP1. So far, unidentified genetic factors are clearly important in the group of sporadic patients who have early disease, and the results point to an early marker. The investigators found that specific phorbol esters reverse these changes in levels and suggest a new physiologically based approach to early treatment (Laperle et al. 2020). Thus, applying cell biology knowledge and methods in this patient group led to the identification of an early marker of the disease, as well as providing clues as to how it might be treated before clinical symptoms become apparent.

## The cell biology of GDNF family ligands and clinical trials

Cell biology approaches have been central to the effort to use glial cell line–derived neurotrophic factor (GDNF) family ligands to treat Parkinson’s disease. GDNF was originally identified in a screen for factors with the capacity to increase the uptake of dopamine in an in vitro assay (Lin et al. 1993). Importantly, GDNF was from the very beginning found to support the survival of midbrain dopaminergic neurons (Lin et al. 1993). Three years later, RET (*re*arranged during *t*ransfection) was identified as a tyrosine kinase transmembrane receptor, which in the presence of the glycosylphosphatidylinositol (GPI)–linked co-receptor GFRa1 (GDNF family receptor alpha 1) mediates GDNF-dependent signaling (Durbec et al. 1996; Jing et al. 1996; Treanor et al. 1996; Trupp et al. 1996). More recently, studies with conditional knock-out mice demonstrated that parvalbumin neuron-derived GDNF maintains dopaminergic neurons in the adult brain (Enterria-Morales et al. 2020). The first trials of GDNF for Parkinson’s disease were very promising. Later blinded control trials were disappointing, not reaching the predetermined outcomes for improvement in motor function (Bartus and Johnson Jr 2017a, b; Gash et al. 2020). Consideration of the problems in the studies as well as the biology of the neurotrophins used can potentially lead to more effective therapies.

## GDNF family ligand signaling

A wide range of knowledge on the ligand/receptor complex has been gathered covering homologs, co-receptors, expression patterns, regulatory elements, biological functions, and results from clinical trials (Airaksinen et al. 2006; Goodman et al. 2014; Ibáñez and Andressoo 2017; Kirkeby and Barker 2019).

To understand what can be done, a brief outline of the signaling complex is essential. As to ligand specificity and receptor complex formation, we now know that four homologous GDNF family ligands can activate RET-signaling via four homologous GPI-linked co-receptors. While GDNF signals via GFRa1 (Treanor et al. 1996), neurturin (NRTN) signals via GFRa2, artemin (ARTN) via GFRa3, and persephin (PSPN) via GFRa4 (for a review see Airaksinen and Saarma 2002). Moreover, evolutionarily distant GDF15/GRAL can also activate RET (Hsu et al. 2017). While all co-receptors can interact with RET, ligand specificity is mediated via differential affinity of the ligands to the co-receptors. However, it is worth noting that cross signaling has also been reported. GDNF can signal via GFRa2 and NRTN via GFRa1 (Buj-Bello et al. 1997; Wang et al. 2000). Furthermore, it is also worth noting that membrane-bound GPI-linked co-receptors can be cleaved off and mediate signaling as soluble co-receptors (Fleming et al. 2015; Wang et al. 2000). Thus, in the presence of soluble co-receptors, GDNF family ligands can active RET also in cells which lack endogenous co-receptor expression. The crystal structures of GDNF/GFRa1 (Parkash et al. 2008), NRTN/GFRa2 (Sandmark et al. 2018), and ARTN/GFRa3 (Wang et al. 2006) complexes have been solved. Even the structures of the full GDNF/GFRa1/RET and NRTN/GFRa2/RET complexes have been resolved using a combination of electron microscopy and low-angle X-ray scattering (Goodman et al. 2014), or cryo-EM (Bigalke et al. 2019).

## Issues in the clinical trials

### Dopamine augmentation versus disease progression

Unfortunately, none of the current medical or surgical therapies for Parkinson’s disease significantly alters the course of the disease (Kalia and Lang 2015). L-Dopa medication and deep brain stimulation (DBS) definitely help symptoms but do little or nothing about the progression of the pathology. Furthermore, even the best current treatments do not restore patients to their normal function. Quantitative measures of voluntary movements demonstrate that by the time mild disease is detected; movement speed has been slowed by 50% or more (Pfann et al. 1998). Treatment with dopaminergic medications makes patients feel and perform better and modestly improves movement speed, but it never returns to normal (Vaillancourt et al. 2004). In later stage disease, DBS subthalamic nucleus stimulation does produce major improvements, i.e., a 50% change in the United Parkinson’s Disease Rating Scale, and it significantly decreases tremor and dyskinesias. However, even in this case, movement speeds do not return to normal (Vaillancourt et al. 2004). Current theories of how subthalamic nucleus works recognize that stimulation does not increase normal functioning but reduces the abnormal signals coming out of the damaged basal ganglia circuits (Chiken and Nambu 2016). Subthalamic nucleus stimulation, like a lesion, regularizes globus pallidus output but does not return the system to its previous functional state (Humphries and Gurney 2012; Humphries et al. 2018).

The course of the disease is marked by a progressive loss of dopaminergic neurons, and thereby, there is an urgent need to identify new treatments which support the survival of these neurons. However, when evaluating the outcome of such trials, it is clear that we need to distinguish between a short-term boost of dopaminergic function providing symptomatic relief, from a long-term protective effect reducing cell death. It is the latter that ultimately will be necessary for a clinically important disease effect.

These biological considerations of Parkinson’s disease mean that human studies have to be powered to find a difference in disease progression and dopamine augmentation which produces only symptomatic relief. As with Alzheimer’s disease, studies may need to be done early in disease development and include longer follow-up. Such study designs are slow, time-consuming, and expensive. Dissecting out changes in symptoms versus the slowing of progression is especially hard when the biological changes due to a neurotrophic factor occur over months.

### Unmet endpoints versus promising observations

The last decade has been a frustrating time for investigators who had envisioned major advances in the treatment of Parkinson’s disease using neurotrophic factors. In spite of very encouraging proof of principle studies, demonstrating a marked improvement in symptoms with NRTN delivered by viral injection, and glial-derived neurotrophic factor (GDNF) infused into the putamen, controlled clinical trial did not achieve a large enough change in motor performance to reach a significant statistical outcome (Gash et al. 2020).

A larger than anticipated placebo response was seen in all the studies. This could be due to placebo effects, bias on the part of raters, or the implantation of a large catheter into the putamen. Postoperative observations indicate that inserting a DBS electrode, which is similar in size to a catheter, into the subthalamic nucleus often results in clinical improvement for days to weeks. The effects of catheter placement and saline infusions are not fully tested. However, the 40-week saline control infusion used in the latest GDNF study patients showed an improvement of off time motor function of 15%. The authors speculate that the implantation and bolus infusions might be the cause of this “placebo effect” (Whone et al. 2019a, b). A study in non-human primates showed a similar improvement in motor function in the control group being infused with saline (Gash et al. 2005).

A number of important positive results from the clinical trials have been pointed out (Kirkeby and Barker 2019). Autopsy findings in patients who died from other causes show dopaminergic nerve fibers streaming towards the catheter positioned in the putamen with infused GDNF. This indicates a strong trophic influence on the dopamine cells. Furthermore, f-Dopa turnover significantly increases in the region surrounding the catheter infused tip. This was true even though clinical responses did not reach significance. In the latest placebo-controlled GDNF trial, all of the treatment measurements during the blinded period and follow-up are better than those of the untreated patients suggesting a true change (Whone et al. 2019b). Analysis of the NRTN study also showed significant improvement in patients treated early in the disease. Other signs of efficacy are found in the two original trials that were done in the USA and UK (Slevin et al. 2007; Gash et al. 2020). Both showed dramatic responses in motor function. In many of these patients, the effect lasted several years after the infusion of GDNF was stopped. Variability in the delivery of the neurotrophic across studies and individual variation within studies may explain why it is been so difficult to demonstrate efficacy.

### Human disease versus animal models

The most straightforward explanation for the failed clinical trials is that GDNF family ligands do not rescue and maintain dopaminergic neurons when used for the human disease, even though they perform well in animal models. Compared with the chemically induced animal disease models, the pathobiology of the human disease is clearly different, and it should not be a surprise that the response is different. In the primate 1-methyl 4-phenyl 1,2,3,6-tetrahydropyridine (MPTP) model, loss of dopamine input is immediate and nearly complete, and the treatment starts as soon as the neurological deficit has stabilized, soon after the lesion. In the human disease, dopaminergic neurons are gradually lost over many decades and clinical diseases are only recognized after 50% or more of neurons are lost. This slow progression leads to a gradual transformation in basal ganglion pathways. The brain is drastically altered morphologically and functionally even before Parkinson’s disease is diagnosed and a therapeutic intervention began. An example of the change is that a lesion, such as a stroke, in the subthalamic nucleus that would cause wild dyskinetic movements in a normal individual, has the opposite effect on a patient with Parkinson’s disease. A lesion in the damaged subthalamic nucleus or globus pallidus internal segment decreases abnormal dyskinetic movements (Pfann et al. 1998). The later the treatment of Parkinson’s disease, the more altered are basal ganglia neurons and their connections and the less likely that pathological will be reversed by therapeutic interventions.

In addition to the animal models which are based on chemical induction, there are animal models which are based on the replication of familial Parkinson’s disease mutations. Many of the symptoms covered by the United Parkinson’s Disease Rating Scale are difficult to evaluate in non-human species. While one animal model may replicate one kind of symptoms of Parkinson’s disease, another animal model may replicate other types of symptoms (Konnova and Swanberg 2018). Even in humans, Parkinson’s disease is a heterogeneous disease with variations in the age of onset, symptoms, and rate of progression. From a cell biology point of view, it is thereby essential but challenging to understand what cell types to target and what types of cellular functions to support.

Different types of neurons are interconnected in a complex network, but also different types of glial cells support essential functions in this network. GDNF has primarily been administered to the putamen to support dopaminergic fibers with cell bodies located in substantia nigra (Ai et al. 2003). GDNF was originally identified as a growth factor supporting mouse embryonic midbrain dopaminergic neurons (Lin et al. 1993), and the expression of its receptors GFRa1 and RET has been verified not only in the putamen of elderly human adults but also in the putamen of patients with Parkinson’s disease (Bäckman et al. 2006). Also, GFRa2 has been detected in human substantia nigra, globus pallidus, and putamen (Runeberg-Roos et al. 2016). However, it would be valuable to know if the expression of the receptor complex differs between patients with varying genetic backgrounds and at various stages of the disease. For GDNF trials, it would be essential to establish that the signaling mediating receptor complex (RET and GFRα receptors) is expressed in the targeted cell types, in all patients selected for trials.

### The importance of accurate drug delivery

Neurotrophin delivery should ideally be to the specific regions having cells that respond and have limited spill over into other regions. Such delivery would maximize positive clinical effects and minimize adverse side effects. In practice, this is difficult to achieve because of the morphology of target regions, somewhat kidney-shaped in the case of the putamen, and the anisotropic nature of brain tissue. The delivery problem is made more difficult by the relatively large size of neurotrophic factors and their limited diffusion in the extracellular space.

The putamen has been the target of choice in Parkinson’s disease because dopaminergic fibers flow into this region where cellular uptake occurs in the widely distributed pre-synaptic dopaminergic projections. Uptake and then retrograde movement allow the factors to be carried to the cell bodies in the substantial nigra in the midbrain. Experiments have looked at distribution from single and multiple needle injection sites in the putamen, slow infusion from catheters, or convection-enhanced delivery which injects large volumes of fluid with enough pressure to cause convective flow in the tissue in the extracellular spaces.

A number of important conclusions can be drawn from the decades of work on this problem. The first and foremost is that the effect of an injected neurotrophin depends on the volume of the target area that is covered. As Gash’s group has clearly demonstrated for GDNF injections in a non-human primate model of Parkinson’s disease, the amount of improvement motor performance measured by a clinical motor rating scale is directly related to the volume covered (Table 1). In a later study using precisely the same implanted drug pump, catheter system and infusion protocol as in the Amgen double-blind study, a fourfold variation in the volume covered, 87 to 369 mm, was observed (Salvatore et al. 2006). The system used for delivery did not work uniformly; it delivered different amounts of drug in each animal.

**Table 1:** Data adopted from Fig. 5A in Gash et al. (2005), showing a significant correlation between improvement score of motor function (non-human primate rating scale, mean of weeks 4–10), and the volume of GDNF distribution in rhesus monkeys with MPTP lesions

GDNF diffusion in rhesus monkeys (mm^3^)	Improvement score of motor function
100	1.5
200	2.7
300	3.5

There is also the problem of scaling up to the much larger human brain. Since the human putamen is 4000 to 5000 mm, five times the size of the monkey, the infusion would be expected to cover only 2 to 9% of its volume (Yin et al. 2009). This certainly calls into question whether or not GDNF helps was tested adequately in the Amgen trial (Fan et al. 2015).

To overcome some of the delivery problems of large therapeutic molecules, convection-enhanced infusions have been employed in both animal and human trials. Normally, distribution of a molecule in the extracellular space is ruled by diffusion down to its concentration gradient and is inversely related to its size. If the molecule is introduced under enough fluid pressure and sufficient volume, flow becomes convective in the extracellular space and can carry both large and small molecules much longer distances in less time (Lonser et al. 2015). Such an approach was used in one of the first open trials of GDNF and produced uniformly positive clinical results (Slevin et al. 2005). Unfortunately, this convection-enhanced protocol was not used for the Amgen controlled trial, and it is likely that the distribution was very limited as the non-human primate study showed (Salvatore et al. 2006).

Another important aspect of any delivery is the diameter of the catheter or needle used. Larger diameters allow fluid to escape along the pathway from the tip of the catheter along the outside of tubing into the CSF spaces over the brain. A further problem with delivery is the infusion of drug near a large blood vessel and its perivascular space. Fluid flow in perivascular spaces is rapid and would dominate as a sink for the molecules that are being delivered near to it. Such vessels cannot be seen on imaging studies and thus cannot be avoided.

The technical ability of the surgeon to correctly position the catheter adds still another level of variability to the trials. Placement precision has improved greatly over time, but small errors could potentially affect delivery. For example, if the delivery is near the adjacent white matter tracks along the putamen which conduct fluid easily, the infusion fluid could be lost and not reach its target site (Linninger et al. 2008). The recent study in Parkinson’s disease use contrast agents to determine the distribution of infused fluid (Whone et al. 2019a). The caveat is that the agent used may distribute differently than the neurotrophic factor.

### Diffusion in brain tissue and modified GDNF family ligands

While the distribution of a neurotrophic factor is critically dependent on where and how it is delivered, it also is dependent on its binding in the extracellular space. Early experiments demonstrated that the distribution of GDNF in the extracellular space is limited by its heparin-binding sites. Co-administration of GDNF and heparin dramatically increase its distribution, but heparin cannot be used in the human clinical trials because of the danger of hemorrhage (Hamilton et al. 2001). The distribution of NRTN in the extracellular space is even more limited, due to its higher heparin-binding properties (Hadaczek et al. 2010). The human putamen is five times bigger than in rhesus monkeys that are tested preclinically for a response to a neurotrophin (Yin et al. 2009). It is critically important to ensure that the infused GDNF protein or virally expressed NRTN protein diffuses readily so that in human trials, enough of the much of the larger putamen is covered.

The structures of the ligands need to be described in order to determine how to achieve a wider distribution in the extracellular space. GDNF family ligands are dimeric ligands with seven S-S bridges (Eigenbrot and Gerber 1997; Parkash et al. 2008; Sandmark et al. 2018; Wang et al. 2006). Six of the bridges are intramolecular, while one is intermolecular, covalently connecting the two monomers. The structure of each monomer has been depicted as a pair of protruding fingers, connected via a hinge (alpha helix). Each monomer interacts with a GFRa receptor via the tips of the fingers (Parkash et al. 2008; Sandmark et al. 2018; Wang et al. 2006). In dimers, there are two pairs of fingers, which drive the dimerization of the receptor complex. While the tips of the fingers have been shown to drive the dimerization of the receptor complex, the alpha helix has been described as a flexible hinge, with no direct interactions with the receptors.

The structure of GDNF is special in the sense that it harbors an extra protrusion at the N-terminus, before the first ligand-binding finger. Heparin-binding sites have been identified in this N-terminal protrusion (Alfano et al. 2007), and to improve the poor bio-distribution of GDNF, an N-terminally truncated version of GDNF was engineered. This variant of GDNF retained the ability to activate the receptor complex, showing a 1.5–1.9-fold increased distribution in rat brains, but was not more efficient than GDNFwt in a 6-OHDA rat model of PD (Smith et al. 2015). Further non-human primate studies with rhesus macaques indicated that the distribution volume of this GDNF variant exceeded the distribution of GDNF wild type by more than 2-fold and that it also increased dopamine turnover similarly to GDNF wildtype (Grondin et al. 2019).

**Fig. 1:**
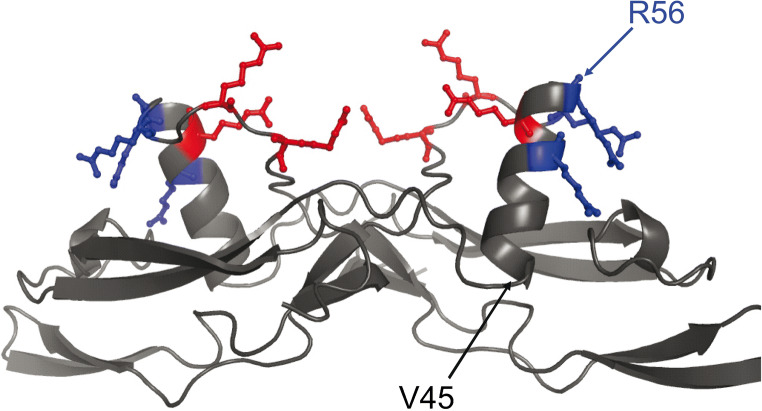
Model of a NRTN dimer, with finger-like structures connected via a helix (heel). The beginning (V45) and end (R56) of the helix are shown with arrows. The location of mutated amino acids is shown in blue and red. With permission from Elsevier, original figure published in Runeberg-Roos et al. (2016)

**Fig. 2:**
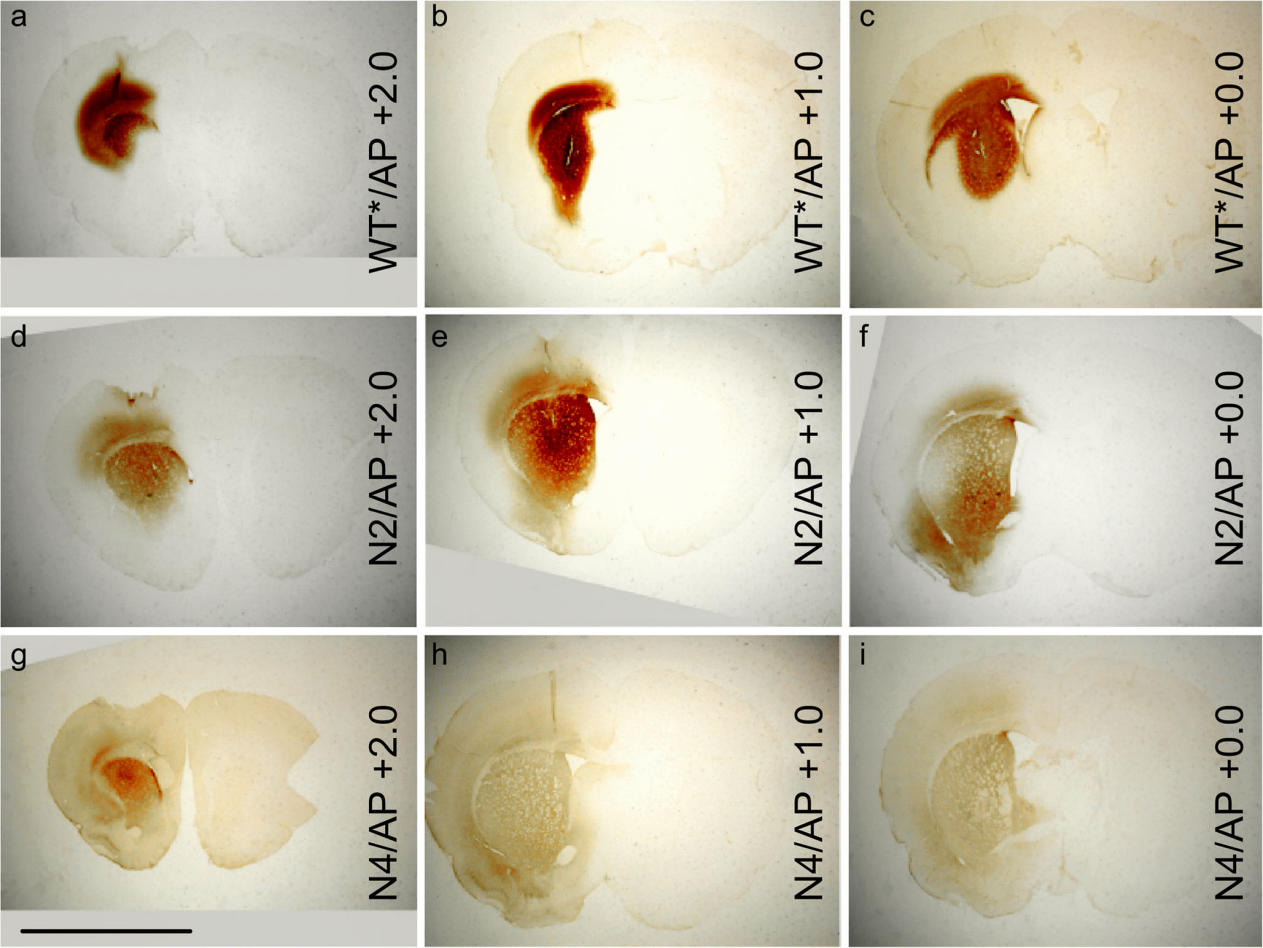
Spreading of WT NRTN in rat brains 24 h after infusion (**a**–**c**), compared with the spreading of the modified NRTN variant N2 (**d**–**f**) and the modified NRTN variant N4 (**g**–**i**). Vertically, the panels in the middle represent sections at the injection site (AP, + 1.0), while sections 1 mm from the injection site are shown to the left (AP, + 2.0) and right (AP, + 0.0). Scale bar 7 mm. With permission from Elsevier, original figure published in Runeberg-Roos et al. (2016)

As NRTN harbors potential heparin-binding sequences within the hinge which connects the two ligand-binding fingers (Fig. 1), it was a natural target for engineering a NRTN variant with reduced binding to heparin (Runeberg-Roos et al. 2016). In three of the engineered NRTN variants, point mutations were introduced in the helix sequence. In the fourth variant, the hinge region of NRTN was substituted with the corresponding hinge region from PSPN. The PSPN hinge was chosen because this GDNF family ligand is highly homologous to NRTN but has a very low affinity for heparin (Alfano et al. 2007; Bespalov et al. 2011). This straightforward engineering approach resulted in a NRTN variant with retained receptor activating capacity, and a 4-fold increased spreading in tissue (Fig. 2). Needless to say, when deleting potential heparin-binding sites from a ligand to make it diffuse more readily, one must make sure that the biological activity, i.e., receptor-binding capacity of the ligand is not impaired. The activity of the NRTN variant N4 was first characterized in cell-based receptor affinity assays, dose-dependent RET-phosphorylation assays, and in vitro survival assays on dopaminergic neurons from mouse midbrains and mouse embryonic kidney cultures. Finally, in a 6-hydroxydopamine rat model of Parkinson’s disease, N4 improved the conditions of the animals more potently than GDNF (Runeberg-Roos et al. 2016).

### Biological activity and prokaryotic versus eukaryotic source of the protein

Clinical trials are naturally planned by medical doctors and pharmacists who have the required solid knowledge on rigorous regulations, complex clinical data, and advanced surgery technology. Although the source of GDNF is just one out of a myriad of components of the trial, its quality is essential for the outcome of the trial. Mammalian GDNF is a glycosylated dimeric protein with seven S-S bridges. In mammalian cells, all secreted proteins are normally subjected to a strict mammalian protein quality control, which ensures a correct folding of the protein. A correct folding is important for both proteolytic stability and biological activity of the secreted protein. The only preparation of GDNF that has been available for clinical trials is by now over two decades old. This version of GDNF was produced without S-S bridges and glycosylation in *Escherichia coli*, using purification procedures that generally include denaturation/renaturation steps. NRTN and GDNF have been shown to be more stable when purified from mammalian cells (Hoane et al. 2000; Piccinini et al. 2013) and more stable ligands naturally have a higher capacity to activate receptor complexes. Using a mammalian source of GDNF for clinical trials would ensure that the trials are done with a high quality of the therapeutically active ingredient.

In the case of viral delivery of GDNF or NRTN, the protein is indeed synthesized and secreted from the patient’s own cells. As with all other technology, the viral delivery of ligands has its own pitfalls. In mammalian cells, endogenous preproNRTN is synthesized at low concentrations with an N-terminal presequence which targets the protein into the endoplasmic reticulum for secretion. The presequence is cleaved off as soon as the protein passes the ER membrane. The role of the prosequence of NRTN has remained elusive, but prosequences are in general thought to assist folding, or sterically hinder the secreted protein from being active before secretion. Prosequences are in general cleaved off before the protein is secreted out of the cell. In the case of NRTN, it is thought that only mature NRTN, lacking the prosequence, is secreted. For viral delivery of NRTN in clinical trials, the endogenous prosequence of NRTN was replaced by the prosequence from NGF (Gasmi et al. 2007).

For unknown reasons, overexpressed preproNRTN is poorly secreted from mammalian cells (Fjord-Larsen et al. 2005) and purified mature NRTN (lacking preprosequences) is a tricky protein which must be handled with great care to avoid aggregation upon buffer exchanges or concentration (unpublished). Therefore, from a protein chemistry point of view, there are good reasons to carefully plan the design of viral delivery of NRTN. A viral vector which drives a high expression of human NRTN may lead to the aggregation of NRTN inside the endoplasmic reticulum, which potentially could hinder NRTN secretion and trigger unfolded protein response, or even cell death. High expression levels of NRTN may also lead to the secretion of partially monomeric NRTN (unpublished), and monomers of NRTN may even inhibit activation of the receptor complex. In clinical trials, the ligand is delivered over long periods of time, so it would be important to make sure that it is secreted as an active dimeric protein, and is not aggregating, subsequently causing endoplasmic reticulum stress and malfunction of the cells which secrete the protein.

### Protein infusion versus viral expression

Neurotrophic factors which could support the survival of neurons, and thereby slow the course of the disease, are proteins which do not pass the BBB. Therefore, their use has so far been dependent on a direct intracranial delivery. This type of delivery is challenging not only from a surgery point of view but also from a protein chemistry point of view. Normally, biological activity of neurotrophic factors is achieved via cellular secretion of very low concentrations of these factors into the extracellular matrix.

GDNF delivery via direct protein infusions has several clear advantages. The dose can be controlled and changed during the course of treatment, the delivery can be stopped at any point, and in theory, even the co-receptor soluble GFRa1 could easily be co-infused. For therapeutic use, GDNF has been dissolved into a synthetic buffer (NaCitrate/NaCl) before infusion into the brain. The pI value of GDNF is high, so a weakly acidic buffer ensures a positive charge of the protein and thereby protects it from aggregation. From a protein chemistry point of view, the injection of the protein into the brain is comparable with a type of buffer exchange. Buffer exchanges are known as hurdles in protein chemistry. They can cause protein aggregation and inactivation, especially if the concentration of the infused protein is high. In part of the clinical trials, the doses of infused GDNF were up to 14 or 30 μg/day (Gill et al. 2003; Slevin et al. 2005), which could create technical difficulties. Systematic characterization of how the activity is affected by different infusion buffers, different concentrations of the infused protein, and various speeds of infusion could solve such problems.

Delivery via viral vectors has the clear advantage that the treatment is achieved via one single surgery. A potential additional advantage is that the expression can be directed to take place only in selected cells, which might mimic a more natural distribution of the ligand inside the tissue. Although the viral vector can not be withdrawn, there is hope that the expression level of the ligand can be regulated via systemic delivery of small-molecule drugs which would keep the expression levels at desired levels (Chtarto et al. 2016). However, it is clear that this type of regulation of the concentration of the secreted protein requires an extra level of technical knowledge, including for instance mastering of leaking promotors as well as the challenge of differential responsivity of different cell types. It should also be kept in mind that part of the inducible vectors drives high expression level when turned on, so even though they would be turned off for periods of time, the protein produced during the boosts of high expression may not be of high quality.

### Low doses versus high doses

Normal concentrations of GDNF in tissue are in the range of 4 pg/mg (Chtarto et al. 2007), and 1–5 ng/ml of GDNF has been shown to induce activation of RET in vitro (Yang et al. 2004; Runeberg-Roos et al. 2016). The average volume of the whole human brain is 1500 cm^3^. This means that if GDNF could diffuse freely and would not be subjected to proteolytic degradation, as low a dose as 1.5–7.5 μg would be enough to activate receptor complexes throughout the whole human brain. Moreover, in the clinical trials, the goal has been to target striatum which is only 7 cm^3^, so for this purpose, only 7–35 ng of GDNF would be needed. In clinical trials, the dose has been more than 10 μg/putamen/day (Gill et al. 2003; Slevin et al. 2005), which means that GDNF has been used in excess. There are probably several reasons behind the use of these high amounts of GDNF: one is that after injection, it attaches to the extracellular matrix, which hinders an efficient spreading from the injection site, and another is that the ligand is degraded by proteolytic enzymes.

However, high local concentrations of the ligand may be contra-productive if the high concentrations induce an aggregation of the protein already upon injection into the tissue. Another more theoretical but still relevant argument against excess amounts of the ligand is based on the available structural data on the ligand/receptor complex. GDNF is a dimeric ligand, which binds two GFRa1 co-receptors and this ternary complex dimerizes and activates two transmembrane tyrosine kinase receptors RET. In theory, a too high concentration of the dimeric ligand can saturate all available GFRa1 co-receptors to the extent that no ternary GDNF/GFRa1 complexes can form (the only formation of trimeric complexes, consisting of dimeric GDNF bound to one GFRa1 co-receptor). In such a situation, the high concentrations of GDNF would de facto inhibit the dimerization and activation of RET.

Therefore, it would be safer to work with low doses of the ligand and instead modify the ligand to achieve both a higher resistance to proteolytic degradation, as well as a lower affinity for the extracellular matrix. In the previously mentioned GDNF variant with reduced heparin-binding, a few amino acid substitutions were indeed introduced with the specific aim to improve the chemical stability of GDNF (Smith et al. 2015). As already mentioned, the N4 variant of NRTN was originally designed to decrease its heparin-binding capacity. Importantly, it is also worth noting that this variant turned out to be less prone to aggregation during purification. In addition, using a kidney in vitro organogenesis assay, we found that its higher biological activity was due to its higher resistance to proteolytic degradation (Runeberg-Roos et al. 2016).

Protein engineering is a powerful tool, which in the context of diabetes has been used to produce both fast- and long-acting insulin variants for the market. In the context of obesity, a long-acting variant of GDF15 was engineered, produced, and successfully tested in animal trials (Xiong et al. 2017). Protein engineering should be considered as an option to improve the effect of the GDNF family ligands for Parkinson’s disease. Two separate groups have already successfully completed protein engineering and subsequent animal trials with variants of GDNF (Smith et al. 2015) or NRTN (Runeberg-Roos et al. 2016). In both cases, the results show improved bio-distribution and increased stability. In the case of the NRTN variant, testing in the MPTP primate model showed enhanced biological and behavioral effects (manuscript in preparation).

### New directions

Making surgical lesions in the basal ganglia structures was the only effective treatment of Parkinson’s disease before the l-Dopa era. Such surgery virtually stopped once l-Dopa was widely used because it was superior and has less side effects. As the progression of the disease despite l-Dopa became clinically apparent, pallidotomy and deep brain stimulation have become an important rescue measure providing several years of symptom relief. The new surgical procedures were made possible by advances in computerized tomography and magnetic resonance imaging–based stereotaxic methods and the availability of pacemaker technology. It is reasonable that advances in cellular biology and physiology will lead to retrials of other treatments that have been sidelined. For example, the transplantation of human fetal dopamine cells initially looked promising. Experience over many years showed that some patients had excellent responses and others did not. (This is very similar to the GDNF trials.) Transplants stopped when a prominent controlled trial showed relatively little long-term clinical improvement and that some patients who improved developed difficult to control dyskinesias, presumably due to too much dopamine. With the knowledge from 30 years of clinical trials and the likelihood that earlier intervention is needed, a new transplant study is being performed on mildly affected Parkinson’s patients (Barker and TRANSEURO consortium 2019). This is intended to set the groundwork for dopamine-producing cells derived from human pluripotent stem cells. Clearly, the ability to produce such dopamine cells from a patient’s own stem cell in the future will reopen the possible effective use of transplantation (Pfisterer et al. 2011).

Likewise, use of cells to deliver GDNF family ligands may expand if such cells can be protected from dying in situ. Coatings and encapsulation have been tried for protecting cells, but so far with little long-term success. However, new neural engineering encapsulation methods as well as more robust cell types are now being investigated (Lindvall and Wahlberg 2008). It is a definite possibility that better results will come from newly designed cell types producing a modified neurotrophic which diffuses well in the extracellular space. Since only nanogram quantities of neurotrophins are needed, small amounts of correctly designed molecules could have a clinically significant effect once the biological problems of protecting the cells and having them survive and secrete are overcome.

Finally, alternate delivery routes are being investigated that enable molecules to get through or around the blood-brain barrier to sites in the brain. Nasal delivery using GDNF encapsulated in a chitosan-coated nanostructured lipid carrier has been shown to improve behavioral as well as histological recovery in a rat model of Parkinson’s disease (Gartziandia et al. 2016). Systemic delivery employing homing peptides has recently been explored. These are interesting peptides which could potentially direct therapeutics and imaging agents to targeted tissues in the body and through the blood-brain barrier to specific regions in the brain (Mann et al. 2017). The fact that exercise is useful in treating the symptoms of Parkinson’s disease (Fisher et al. 2013) suggests that some endogenous molecule released from muscle activity penetrates the blood-brain barrier to change the brain’s pathophysiology. An increase in BDNF in the brain after exercise indicates that neurotrophic factors may be involved in this improvement (Mitchell et al. 2010). Current studies are testing whether exercise can actually slow the progression of the disease.

## Conclusions

Taken together, GDNF family ligands have not yet been fully perfected and problems of biological activity, dosage, distribution, and the method of delivery need to be carefully reevaluated. New trials have to be designed to find the optimal dosage and be able to follow patients long enough and with the right protocols to separate symptomatic relief from a disease-modifying effect. These are difficult tasks but are warranted because of strong signs in animal and clinical studies that indicate biologically effects which could well be useful in treating this increasingly prevalent and debilitating, deadly disease.
